# Credible Threat: Perceptions of Pandemic Coronavirus, Climate Change and the Morality and Management of Global Risks

**DOI:** 10.3389/fpsyg.2020.578562

**Published:** 2020-10-30

**Authors:** Ann Bostrom, Gisela Böhm, Adam L. Hayes, Robert E. O’Connor

**Affiliations:** ^1^Daniel J. Evans School of Public Policy & Governance, University of Washington, Seattle, WA, United States; ^2^Department of Psychosocial Science, University of Bergen, Bergen, Norway; ^3^Department of Psychology, Inland Norway University of Applied Sciences, Lillehammer, Norway; ^4^School of Marine and Environmental Affairs, University of Washington, Seattle, WA, United States; ^5^Division of Social and Economic Sciences, National Science Foundation, Arlington, VA, United States

**Keywords:** pandemic, coronavirus, climate change, risk perception, risk management, worry budget

## Abstract

Prior research suggests that the pandemic coronavirus pushes all the “hot spots” for risk perceptions, yet both governments and populations have varied in their responses. As the economic impacts of the pandemic have become salient, governments have begun to slash their budgets for mitigating other global risks, including climate change, likely imposing increased future costs from those risks. Risk analysts have long argued that global environmental and health risks are inseparable at some level, and must ultimately be managed systemically, to effectively increase safety and welfare. In contrast, it has been suggested that we have worry budgets, in which one risk crowds out another. “In the wild,” our problem-solving strategies are often lexicographic; we seek and assess potential solutions one at a time, even one attribute at a time, rather than conducting integrated risk assessments. In a U.S. national survey experiment in which participants were randomly assigned to coronavirus or climate change surveys (*N* = 3203) we assess risk perceptions, and whether risk perception “hot spots” are driving policy preferences, within and across these global risks. Striking parallels emerge between the two. Both risks are perceived as highly threatening, inequitably distributed, and not particularly controllable. People see themselves as somewhat informed about both risks and have moral concerns about both. In contrast, climate change is seen as better understood by science than is pandemic coronavirus. Further, individuals think they can contribute more to slowing or stopping pandemic coronavirus than climate change, and have a greater moral responsibility to do so. Survey assignment influences policy preferences, with higher support for policies to control pandemic coronavirus in pandemic coronavirus surveys, and higher support for policies to control climate change risks in climate change surveys. Across all surveys, age groups, and policies to control either climate change or pandemic coronavirus risks, support is highest for funding research on vaccines against pandemic diseases, which is the only policy that achieves majority support in both surveys. Findings bolster both the finite worry budget hypothesis and the hypothesis that supporters of policies to confront one threat are disproportionately likely also to support policies to confront the other threat.

## Introduction

Rarely has humanity faced two powerful environmental threats to global well-being simultaneously. For decades scholars have been studying how people perceive climate change and what they are doing, would do, and want their governments to do to address the threat posed by climate change to themselves as individuals, to their nations, and to global well-being (e.g., Fischhoff and Furby, 1983; Löfstedt, 1991; O’Connor et al., 1999; Böhm and Pfister, 2001; Leiserowitz, 2005; Bostrom et al., 2012; Lee et al., 2015). With the emergence of the pandemic coronavirus at the close of 2019, scholars are asking how people are protecting themselves and what they want their governments to do to address the threat posed by pandemic coronavirus. Both lines of research are providing useful information on the political, psychological, and social determinants of attitudes toward these risks. Little research, however, compares the two threats in the public mind or looks at how the presence of a second potentially calamitous threat influences attitudes toward the other threat.

This paper reports results from an April 2020 survey of 3,203 respondents in the United States to identify the fundamental similarities and differences in how the public understands these threats and how these views of the nature of the threat influence the level of concern and willingness to act in the public interest. Policy preferences flow from levels of dread (Fischhoff et al., 1978), but also from efficacy judgments (both for personal actions and government policies; Bostrom et al., 2019) and moral responsibility assessments (Doran et al., 2019).

The presence of two powerful threats at the same time may influence what people are willing to support differently than if there were only one threat. One logical hypothesis is that people who are deeply concerned about addressing either climate change or the coronavirus pandemic are part of a cultural community that is likely to view the threat as systemic and needing a coherent institutional response. The idea is that people who demand a strong governmental response to the coronavirus pandemic threat are more likely also to demand a strong governmental response to the threat from climate change (and vice-versa) because they understand that these sorts of threats require a strong governmental response. There is a “crowding-in” phenomenon by which recognition that one of the threats needs a strong centralized policy response makes an individual more likely to perceive that the other threat also needs a strong centralized policy response. In contrast, to “crowding-in” that leads to systemic thinking in general and recognition that the world and national communities must act, there is a “crowding-out” hypothesis that argues that people have a “worry budget” so that great concern for one threat reduces concern for and willingness to confront the other threat (Linville and Fischer, 1991; Weber, 2006; Huh et al., 2016). The idea is that people can devote only so much energy to caring about and addressing problems, so that the increase in concern for pandemics would limit concern for climate change (and vice versa).

In summary, after describing the materials, procedures and methods of data acquisition and treatment, this paper compares the psychometrics for each threat, identifies the determinants of support for policies to address the threat, assesses the finite pool of worry thesis, and concludes with a discussion of the significance of the findings.

## Materials and Methods

### Sampling Procedure

The study is a paywall-intercept (also called “survey wall”) survey experiment conducted in the United States through Google Surveys publisher network, to achieve a representative sample of internet users. Google Surveys samples tens of millions of internet users daily using a “river sampling” or “web intercept” sampling approach, through a network of publishers on over 1,500+ sites publishing a variety of content, including 74% News, 5% Reference, 4% Arts and Entertainment, and 17% other (McDonald et al., 2012; Sostek and Slatkin, 2018). Google Surveys pays these publishers. Surveys are kept extremely short, up to a maximum of 10 questions, with formats restricted to minimize response burdens. All responses are anonymous. The surveys are offered by publishers to internet users, who can choose to pay for accessing the publisher’s content instead of answering questions, or can skip the survey. Internet users are selected through a computer-algorithm-driven stratified-sampling process to create an internet-user sample that matches the national internet-using population age, gender and location. Users cannot opt into surveys; they are assigned a random survey from those available (Keeter and Christian, 2012). For paywall intercept surveys run on the Google survey platform in the first half of 2018, the response rate was 25% (Sostek and Slatkin, 2018). Although Google Surveys publisher network does not offer a population random sample, comparative analyses have concluded that it provides a sample of adult internet users that is as representative as others available, appropriate and sufficiently accurate for survey experiments (Keeter and Christian, 2012; McDonald et al., 2012; Santoso et al., 2016), and that the platform is useful given its affordability and ease of survey implementation (e.g., Tanenbaum et al., 2013). In comparative studies Google Surveys samples have been found to be highly representative of the internet-user population in the United States, for example including more conservatives (40%) than a Pew Research survey sample (36%) (Keeter and Christian, 2012, p. 9).

The study was reviewed by the University of Washington Human Subjects Division and determined to be exempt from federal human subjects regulations (IRB ID STUDY00009946). Data collection took place in mid-April 2020 (April 12–17, 2020).

### Participants

A total of *N* = 3203 U.S. adults completed all 10 questions in the survey block they were offered (see below), out of 4,570 who answered the first question. The drop-off rate (after the first question) was 29.3% on average for the pandemic survey blocks, and 30.5% on average for the climate survey blocks. Age and gender were inferred by Google; for the 818 participants who had opted out in the Ads setting (which applies to Google survey as well) age and gender are unknown. The estimated distribution across the age categories 18–24, 25–34, 35–44, 45–54, 55–64, 65+ years was 243, 371, 422, 383, 478, and 488, respectively. Gender was not inferred for 714 participants; 1,295 participants identified as female, 1,194 as male. For the publisher network samples, Google estimates response biases for each survey, comparing age, gender, and region to provide weights for a representative sample, and reports the bias in the sample as Root Mean Square Error estimated across these characteristics, for each question. RMSE varied from 2 to 4.4% for questions in our surveys. In general our samples slightly overestimate those aged 55–64 and 65+, and those living in the Midwestern U.S. Because weights are not calculated for those opting out, weighted samples are much smaller. Our sensitivity analyses comparing results on analyses conducted with weighted versus unweighted data revealed no noteworthy differences in results (e.g., differences in percentages supporting policies between the weighted and unweighted data were in the tenth of a percent range), for which reason we report analyses using unweighted data.

### Materials

The questionnaire consisted of measures on psychometric judgments, policy preferences, and political orientation. Age and gender were inferred for all participants by Google.

**FIGURE 1:**
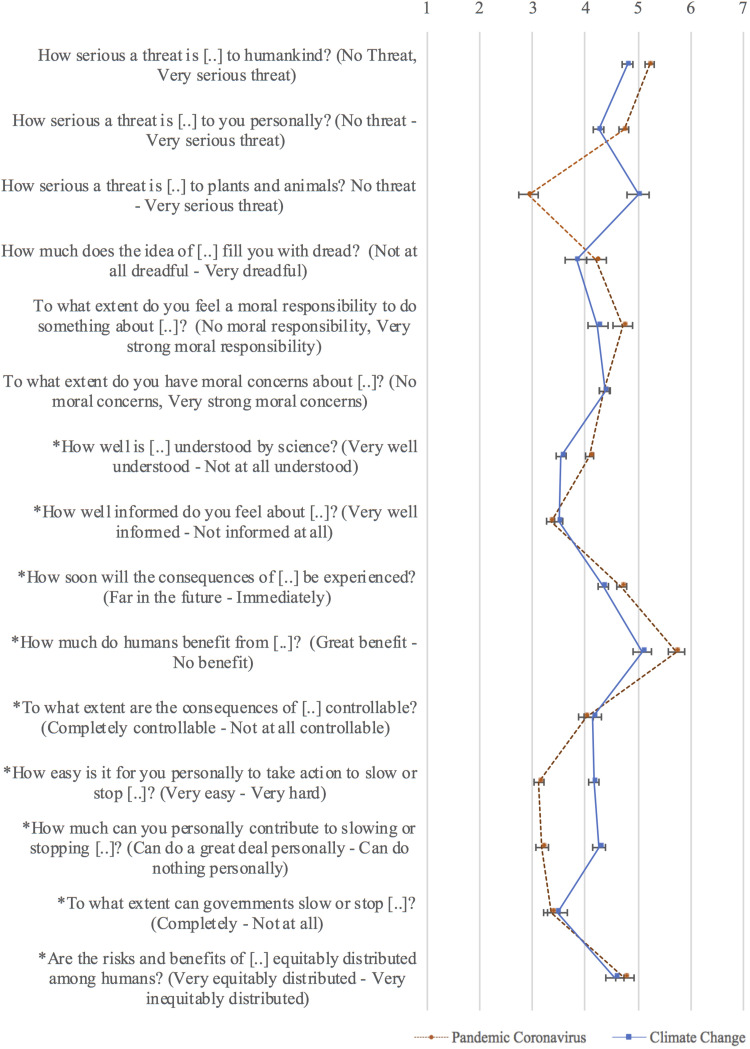
Average psychometric risk ratings from the raw data (no imputations included), by risk, with 95% confidence intervals for the means. Sample sizes vary from 400 to 1,601 per mean, as seven of these survey questions were presented in only one block, one in two blocks, one in three, and six in all four blocks. *Indicates that the item has been reverse coded for purposes of this figure, so that the response scale is in the direction indicated in parentheses; this way higher numbers imply higher perceived risk consistently for all items.

The total set of survey items included 15 psychometric risk judgments adapted from prior risk perception research on what is often referred to in risk research as the psychometric paradigm (Slovic, 1987; Bostrom et al., 2012, 2020). Each item has a seven-point rating scale with labeled endpoints. The psychometric judgments tapped into the following facets of perceived risk: threat and dread, known risk, morality, controllability and efficacy, and human benefits. Depending on the experimental condition (i.e., version of the survey), respondents provided psychometric judgments with respect to either global climate change or the pandemic coronavirus. Figure 1 and Table 1 list the complete wording and response scale labels.

**TABLE 1:** Psychometric judgments and factor models used in confirmatory factor analyses.

		Assignment of variables to factors		
No.	Item	Model 1	Model 2	Model 3
**Threat and dread**				
1	How serious a threat is < *climate change/pandemic coronavirus* > to humankind? (No Threat—Very serious threat)	Factor 1	Factor 1	Factor 1
3	How serious a threat is < *climate change/pandemic coronavirus* > to you personally? (No threat—Very serious threat)	Factor 1	Factor 1	Factor 1
4	How serious a threat is < *climate change/pandemic coronavirus* > to plants and animals? (No threat—Very serious threat)	Factor 1	Factor 1	Factor 1
7	How much does the idea of < *climate change/pandemic coronavirus* > fill you with dread? (Not at all dreadful—Very dreadful)	Factor 1	Factor 1	Factor 1
**Morality**				
14	To what extent do you have moral concerns about < *climate change/pandemic coronavirus* > ? (No moral concerns—Very strong moral concerns)	Factor 1	Factor 2	
13	To what extent do you feel a moral responsibility to do something about < *climate change/pandemic coronavirus* > *?* (No moral responsibility—Very strong moral responsibility)	Factor 1	Factor 2	
10	Are the risks and benefits of < *climate change/pandemic coronavirus* > equitably distributed among humans? (Very inequitably distributed—Very equitably distributed)	Factor 1	Factor 2	
**Known risk**				
2	How well is < *climate change/pandemic coronavirus* > understood by science? (Not at all understood—Very well understood)	Factor 2	Factor 3	Factor 2
11	How well informed do you feel about < *climate change/pandemic coronavirus* > ? (Not informed at all—Very well informed)	Factor 2	Factor 3	Factor 2
12	How soon will the consequences of < *climate change/pandemic coronavirus* > be experienced? (Immediately—Far in the future)	Factor 2	Factor 3	Factor 2
**Controllability and efficacy**				
6	To what extent are the consequences of < *climate change/pandemic coronavirus* > controllable? (Not at all controllable—Completely controllable)	Factor 2	Factor 4	
8	How easy is it for you personally to take action to slow or stop < *climate change/pandemic coronavirus* > ? (Very hard—Very easy)	Factor 2	Factor 4	
8B	How much can you personally contribute to slowing or stopping < *climate change/pandemic coronavirus* > ? (Can do nothing personally—Can do a great deal personally)	Factor 2	Factor 4	
9	To what extent can governments slow or stop < *climate change/pandemic coronavirus* > ? (Not at all—Completely)	Factor 2	Factor 4	
**Human benefits**				
5	How much do humans benefit from < *climate change/pandemic coronavirus* > ? (No benefit—Great benefit)	Factor 2	Factor 4	

*Depending on their survey context condition, participants responded to either the climate change or the pandemic coronavirus version of each item. Endpoint labels of response scales are given in parentheses. Models 1 and 2 use all psychometric items; Model 3 uses only seven of them.*

**FIGURE 2:**
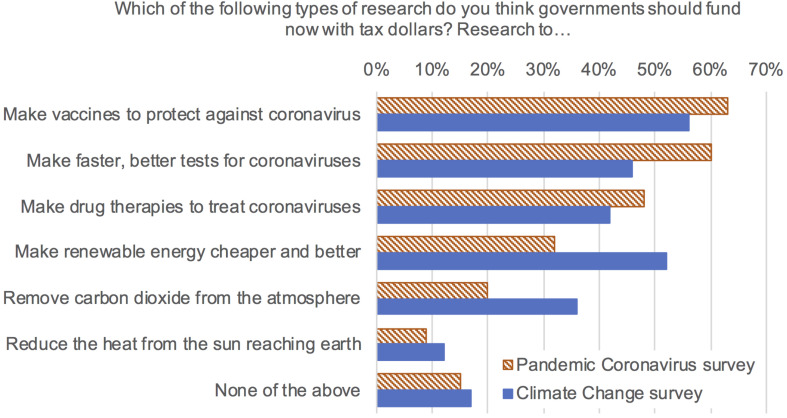
Between survey comparison of percentage of respondents selecting to support each category of research (*N* = 800, 400 per survey).

Preferences with regard to supporting or not supporting each of six policies were posed to all participants in block D in a check-all-that apply survey question (response order randomized, with an explicit “None of the above” option presented last). Three of these referred to policies regarding climate change, the other three to policies addressing the coronavirus pandemic. For each risk issue, we selected a policy that is popular (e.g., research on renewable energy, to address climate change; Howe et al., 2015), and a policy associated with some contention or disagreement (e.g., funding research on solar radiation management to address climate change). Figure 2 provides the exact wording for the six policies and percentages supporting each of them.

Political orientation was measured with the prompt “Would you describe yourself as” and a seven-point rating scale with the verbal endpoint anchors “Extremely liberal” and “Extremely conservative.”

### Survey Procedures

In order to fit the constraints of Google’s survey platform, which allows a maximum of 10 questions per survey, we implemented a sparse matrix design which randomized respondents to one of eight distinct blocks, four on pandemic coronavirus, four on climate change. From the set of measures on psychometric judgments and policy support, we grouped questions into four distinct blocks of 10 questions each, with a few core questions asked across all blocks, following existing guidance for sparse matrix designs (e.g., Rhemtulla and Hancock, 2016). The order of the questions varied by block, but each block was exactly the same across the two risks (pandemic coronavirus and climate change). The check-all-that-apply survey question about policy preferences appeared only in block D and was presented as the last question in that block. Participants were randomly assigned to answer survey items from a single block.

### Imputation

The sparse matrix survey design results in systematically missing data as respondents could not answer the items that were not included in their randomly assigned survey block. We resolved this issue by using multiple imputation to construct a complete dataset that could then be analyzed. In particular, we constructed 100 imputed datasets using the Amelia package in R (Honaker et al., 2019). The Amelia algorithm assumes data are jointly multivariate normal, and uses a bootstrapped Expectation-Maximization (EM)-algorithm to generate complete datasets from the posterior distributions (Honaker and King, 2010). While the missing survey data contain dichotomous and seven-point Likert items that do not match the assumption of multivariate normality, research has shown that this imputation method works nearly as well for handling these data types as imputation methods that are more specialized but also less robust (Kropko et al., 2014). We imputed missing data only if they were missing due to the survey block randomization, fulfilling the requirement that imputed data be missing at random (Rubin, 1976).

The imputation procedure incorporated all survey data, including all psychometric judgments, policy support, and political orientation, as well as risk comparison questions that asked respondents how more familiar risks compare to management of coronavirus and climate change, respectively, and a question asking participants to rate how similar managing pandemic coronavirus is to managing climate change (or vice versa, for the climate change survey). The imputation procedures also included demographic information from Google, such as gender, age category, and categorical geographic information. Prior to imputation we centered all non-binary variables and dichotomized respondents’ categorical geographic information which we included in the imputation only if at least 10 respondents shared a particular location.

We used the mean variance-covariance matrix across all 100 imputations to conduct the principal components analysis (Van Ginkel, 2010; Van Ginkel and Kroonenberg, 2014), and the confirmatory factor analyses.

The regression analyses were estimated by bootstrapping each regression model equally across all imputations and limiting the regression to fit only the observations for which the dependent variable is not imputed. We imputed age and gender data for any respondents for which Google was unable to infer this information. We included the imputed gender values in the regression, but only included the non-imputed age due to apparently poor quality of the imputed age values. Our results reported in the following section are not sensitive to these choices. Nor are the results substantially different if we round the ordinal categorical imputations or restrict the imputed data to the initial data range.

## Results

We report the results in three sections. First, we report results concerning the psychometric scales. A description of the profiles of climate change and coronavirus pandemic on the psychometric scales is followed by factor analyses that were undertaken in order to inspect the correlational structure of the psychometric items. The last two sections of the results then focus on testing the worry budget versus crowding-in hypotheses more specifically. This is first done by reporting regression analyses to account for policy preferences then by analyzing the effects of the survey context (climate change versus pandemic coronavirus) on perceived risk and policy preferences.

### Psychometric Judgments

#### Profiles of Climate Change and Pandemic Coronavirus

Participants rated pandemic coronavirus as slightly more dreadful and threatening to humankind and to themselves personally than climate change, but far less threatening to plants and animals, as might be expected (Figure 1). Despite feeling almost equally well informed about both risks, participants rated pandemic coronavirus as less understood by science than climate change. They also judged it harder to take action and to personally contribute to slowing or stopping climate change than to pandemic coronavirus, and felt a greater moral responsibility to do something about pandemic coronavirus. Nevertheless, they reported similar levels of moral concerns across both risks.

### Dimensional Structure of Psychometric Judgments

In order to investigate the dimensional structure of the psychometric judgments, we conducted principal component and confirmatory factor analyses. For all factor analyses we used the R statistical environment (R Core Team, 2020).

For comparative purposes, we started by following the procedures introduced in risk research by the psychometric paradigm in the 1970s and 1980s (e.g., Slovic, 1987), which entail conducting exploratory principal component analyses (PCA) with varimax rotation. We conducted these analyses separately for each of the two risk issues pandemic coronavirus and climate change. Based on the scree test and Kaiser criterion (Eigenvalue of at least 1.0), we inspected the 2-, 3-, and 4-factor solutions. The results turned out to be unsatisfactory. While the first factor could generally be interpreted as a threat/dread and morality factor, the other items did not form a clearly interpretable factor structure and showed substantial cross-loadings. Also, in the case of climate change, the known risk items did not form a separate factor, as has been found previously in the psychometric literature, but instead loaded on the first factor together with threat/dread and morality (see online supplement for additional details).

We therefore proceeded by trying to identify a consistent factor structure in a confirmatory rather than exploratory manner. We derived three factor models from the literature (see Table 1), two from empirical work by Bostrom et al. (2020), who used a set of psychometric items almost identical to ours, and one from the seminal work by Slovic and colleagues on the psychometric paradigm (specifically, from Slovic, 1987).

Similar to our study, Bostrom et al. (2020) measured perceived risk concerning climate change and pandemic influenza (within-subjects, in contrast to our between-subjects design) on psychometric items that correspond to 12 of our 15 items. They report (a) a two-factor solution that they computed separately for climate change and pandemic influenza and which replicated across the two risks, and (b) a four-factor solution that was computed analyzing both risks together. Models 1 and 2 (Table 1) were specified according to Bostrom et al.’s two- and four-factor solutions, respectively. These two models use all 15 items. The three items that our questionnaire included in addition to Bostrom et al.’s twelve items were allocated to the factor that matched them conceptually.

A robust finding in the literature on the psychometric paradigm is that two factors have emerged across various risk domains and respondent populations: Dread Risk and Known Risk. We specified Model 3 (Table 1) by selecting the marker items of these two traditional factors from our items, resulting in a two-factor model using seven of our items.

We estimated all models separately for pandemic coronavirus and climate change, and with both orthogonal and correlated factors. Table 2 shows the goodness-of-fit indices of the models (two models could not be estimated, see note to Table 2). The only model that approached acceptable fit measures was Model 3. For climate change, it could only be estimated with correlated factors; for pandemic coronavirus, the fit is better with correlated than with orthogonal factors. We therefore display loadings only for Model 3 with correlated factors (see Table 3).

**TABLE 2:** Goodness-of-Fit indicators of confirmatory factor analyses.

Model	χ*^2^*	*df*	χ*^2^/df*	GFI	RMSEA
**Climate change (*n* = 1,601)**					
Model 1 (orthogonal)	4747.56***	90	52.75	0.74	0.18
Model 1 (correlated)	2729.31***	89	30.67	0.85	0.14
Model 2 (orthogonal)	7058.52***	90	78.43	0.61	0.22
Model 3 (correlated)	443.60***	13	34.12	0.95	0.14
**Coronavirus pandemic (*n* = 1,602)**					
Model 1 (orthogonal)	2118.49***	90	23.54	0.73	0.12
Model 1 (correlated)	1694.97***	89	19.04	0.79	0.11
Model 2 (orthogonal)	3066.46***	90	30.07	0.61	0.14
Model 2 (correlated)	1365.10***	84	16.25	0.83	0.10
Model 3 (orthogonal)	287.50***	14	20.54	0.92	0.11
Model 3 (correlated)	144.49***	13	11.11	0.96	0.08

*** < 0.001. orthogonal: orthogonal factors. correlated: correlated factors. For climate change, Model 2 with correlated factors and Model 3 with orthogonal factors did not converge. Models 1 and 2 use all psychometric items; Model 3 uses only seven of them.

**TABLE 3:** Unstandardized loadings (standard errors) and standardized loadings for Model 3 (correlated) confirmatory factor analyses of climate change (*n* = 1,601) and coronavirus pandemic (*n* = 1,602).

	Climate change				Coronavirus pandemic			
Item	Threat/Dread		(Un)Known risk		Threat/Dread		(Un)Known risk	
	Unstandardized	Standardized	Unstandardized	Standardized	Unstandardized	Standardized	Unstandardized	Standardized
Threat to humankind	1.00 (*–*)	0.93 (0.02)			1.00 (*–*)	0.85 (0.02)		
Personal threat	0.97 (0.02)	0.91 (02)			1.09 (0.03)	0.87 (0.02)		
Threat to animals, plants	1.02 (0.01)	0.94 (0.02)			0.79 (0.03)	0.61 (0.02)		
Dread	0.92 (0.02)	0.83 (0.02)			0.94 (0.03)	0.73 (0.02)		
Understood by science			1.00 (*–*)	−0.96 (0.03)			1.00 (*–*)	0.65 (0.04)
Well informed			0.41 (0.03)	−0.46 (0.03)			1.13 (0.10)	0.73 (0.04)
Delay of consequences			−0.31 (0.03)	0.29 (0.03)			0.50 (0.06)	0.26 (0.03)

*See Table 1 for complete item wording. Dashes (–) indicate that standard error was not estimated. GFI = 0.95 (climate change), 0.96 (coronavirus pandemic); RMSE = 0.14 (climate change), 0.08 (coronavirus pandemic). χ^2^(13) = 443.60, p < 0.001 for climate change; χ^2^(13) = 144.49, p < 0.001 for coronavirus pandemic. The correlation between Threat/Dread and Known Risk latent variables is −0.78 for climate change and.39 for coronavirus pandemic.*

In sum, we find supportive evidence for the two traditional psychometric factors: Dread and Known Risk in their pure form, that is, using only marker items for each of these two factors. For the remaining items, we could not identify a consistent factorial structure. For Model 3 the structure of the Known Risk factor differs for climate change and pandemic coronavirus (Table 3). For climate change, the loadings of the extent to which the risk issue is understood by science and of how well informed the respondent feels, on the one hand, and of how delayed the consequences are perceived to be, on the other hand, have different signs. That is, respondents believe that the risk issue is less understood by science and feel less informed themselves the more delayed they perceive the consequences of climate change to be. For pandemic coronavirus, in contrast, the loadings of these three items on the Known Risk factor have the same sign. Hence, for pandemic coronavirus, respondents believe that the risk issue is better understood by science and feel better informed the more delayed the perceived consequences are. One potential explanation of this difference may lie in the fact that climate change and pandemic coronavirus differ in familiarity. Climate change is by now an “old” risk and people may believe that science knows a great deal about it. Temporal delay of consequences may then be associated with greater uncertainty of predictions. Pandemic coronavirus, in contrast, is a new risk that just emerged a couple of months before our survey was administered. Albeit with concerted and prolific research efforts, science had just started to investigate the virus at the time of the survey. In such a situation, people may regard delayed consequences as providing the opportunity for science to accumulate more insights.

### Predicting Policy Support

The numbers of policies supported by survey context and risk type are reported in Table 4. Figure 2 shows the percentage of each sample supporting each policy.

**TABLE 4:** Percentage of survey participants supporting none, 1, 2 or all 3 research policies*, for each risk and by risk survey.

	Pandemic research		Climate research	
Number of policies supported	Pandemic survey (% of 400)	Climate survey (% of 400)	Pandemic survey (% of 400)	Climate survey (% of 400)
0	20.3	33.3	64.3	41
1	26	21.5	16.8	28.2
2	15.5	12.3	12.8	21
3	38.3	33	6.3	9.8
	100	100	100	100

*Specific policies are itemized in Figure 2.

To examine whether risk perceptions as measured on psychometric scales are associated with risk policy support, we created additive scales corresponding to the factors we hypothesized. Based on the factor analyses reported in section “Dimensional structure of psychometric judgments,” we calculate the average of four items to create a Threat scale (Cronbach’s alpha 0.91): threat to humankind, personal threat, threat to plants and animals, and dread. The Known Risk factor that emerged from confirmatory factor analysis was not reliable by common standards, for which reason we calculate the average of two items (Cronbach’s alpha 0.62)–understood by science and well informed–to represent this factor in the regression analyses. Averaging the items measuring moral responsibility and moral concerns produces a Moral scale with a Cronbach’s alpha of 0.86. Efficacy is measured with the average of four items (Cronbach’s alpha 0.82): ease of personal action, personal contribution to slowing or stopping the risk, the extent to which government can slow or stop the risk, and the controllability of the risk.

Ordinal probit models predicting the number of policies supported were estimated separately for each risk. Consistently across both coronavirus and climate change risks, greater perceived threat was associated with greater support for government funding research on addressing pandemic disease and climate change (Tables 5, 6 show the mean coefficients estimated across 100 imputed datasets for Block D of each risk, in which no dependent variable data are imputed). In the climate change survey, both higher perceived threat and greater moral concerns correlate with supporting more investments in government funding for research to address climate change, controlling for all else. Although the estimated mean coefficient for the Moral scale does not quite rise to standard levels of significance, the coefficient magnitude is relatively large compared to other coefficients in the model.

**TABLE 5:** Model to predict the number of coronavirus-related policies that a respondent supports.

	Mean estimate	2.5th percentile	97.5th percentile
**Threshold**			
[PandemicResearch = 0| 1]	–0.803		
[PandemicResearch = 1| 2]	0.002		
[PandemicResearch = 2| 3]	0.421		
**Location**			
ThreatScale	0.201	0.097	0.307
KnownScale	0.054	–0.059	0.166
MoralScale	0.002	–0.098	0.106
EfficacyScale	0.008	–0.149	0.160
Conservative	–0.034	–0.138	0.066
Female	–0.092	–0.356	0.171
Age	0.055	–0.018	0.130
Age = Unknown	0.180	–0.103	0.466

*Ordered Probit. Mean Log Likelihood = −507. Mean AIC = 1,036. Nagelkerke pseudo-R^2^ = 0.11.*

**TABLE 6:** Model to predict the number of climate change policies that a respondent supports.

	Mean estimate	2.5th percentile	97.5th percentile
**Threshold**			
[ClimateResearch = 0| 1]	0.005		
[ClimateResearch = 1| 2]	0.953		
[ClimateResearch = 2| 3]	1.926		
**Location**			
Threat Scale	0.230	0.081	0.374
KnownScale	0.061	–0.063	0.187
MoralScale	0.131	–0.027	0.297
EfficacyScale	–0.054	–0.239	0.120
Conservative	–0.008	–0.117	0.095
Female	0.100	–0.193	0.403
Age	0.002	–0.08	0.082
Age = Unknown	0.184	–0.099	0.457

*Ordered Probit. Mean Log Likelihood = −439. Mean AIC = 899. Nagelkerke pseudo-R^2^ = 0.30.*

For the two most popular policies, research on vaccines and research on renewable energy, binary probit regressions were also estimated for each of the 100 imputed datasets for each risk, restricted to Block D. Here again, perceived Threat from pandemic coronavirus is positively associated with being more likely to support research on vaccines for pandemic diseases, and perceived Threat from climate change is positively associated with favoring government support for research on renewable energy (Tables 7, 8).

**TABLE 7:** Binary probit regression predicting support for vaccines.

	Mean estimate	2.5th percentile	97.5th percentile
ThreatScale	0.251	0.130	0.379
KnownScale	0.104	–0.024	0.236
MoralScale	0.000	–0.126	0.128
EfficacyScale	–0.061	–0.254	0.129
Conservative	–0.034	–0.158	0.092
Female	–0.062	–0.380	0.247
Age	0.013	–0.076	0.103
Age = Unknown	0.059	–0.266	0.389
Constant	0.297	0.069	0.537

*Dependent variable: “Which of the following types of research do you think governments should fund now with tax dollars? Research to… make vaccines against pandemic diseases” (1 = Yes, 0 = No, N = 400). Mean Log Likelihood = −240, Nagelkerke pseudo-R^2^ = 0.11. Bootstrapped confidence intervals.*

**TABLE 8:** Binary probit regression predicting support for renewable energy.

	Mean estimate	2.5th percentile	97.5th percentile
ThreatScale	0.173	0.004	0.347
KnownScale	0.085	–0.059	0.232
MoralScale	0.163	–0.018	0.352
EfficacyScale	–0.050	–0.277	0.168
Conservative	–0.010	–0.139	0.116
Female	0.076	–0.289	0.440
Age	0.057	–0.039	0.153
Age = Unknown	0.161	–0.175	0.508
Constant	–0.242	–0.537	0.036

*Dependent variable: “Which of the following types of research do you think governments should fund now with tax dollars? Research to… make renewable energy cheaper and better (1 = Yes, 0 = No, N = 400). Mean Log Likelihood = −223, Nagelkerke pseudo-R^2^ = 0.23. Bootstrapped confidence intervals*

### Effects of Survey Context

Policy preferences are stronger within a same-topic context; in other words, the average number of pandemic disease mitigation research policies supported in the pandemic coronavirus survey is higher than the average number of pandemic disease mitigation policies supported in the climate change survey. Similarly, the average number of climate change policies supported in the climate change survey is higher than the average number of climate change policies supported in the pandemic coronavirus survey context. Additionally, overall, respondents support more research funded by tax dollars to address pandemic diseases than they do to address climate change, controlling for political orientation (Tables 4, 9). These results support a “worry budget” narrative, although support for research on risk mitigation of pandemic diseases does not completely crowd out support for research on approaches to reducing the risks of climate change. In fact, the number of policies supported for research to mitigate the risks of pandemic diseases is positively correlated with the number of policies supported for research to mitigate the risks of climate change (*r* = 0.407, *p* < 0.001 partial correlation, controlling for political orientation).

**TABLE 9:** One-way ANCOVA of the survey context effect and differences between risks, controlling for political orientation.

			95% confidence interval for mean	
	N	Mean number of policies supported	Lower bound	Upper bound
**Pandemic research**				
Pandemic survey	400	1.72	1.60	1.83
Climate survey	400	1.45	1.33	1.57
Total	800	1.58	1.50	1.67
**Climate research**				
Pandemic survey	400	0.61	0.52	0.70
Climate survey	400	1.00	0.90	1.09
Total	800	0.80	0.73	0.87

*Political orientation [F(2, 796) = 14.385, p < 0.001], mean difference between survey types [F(2, 796) = 28.612, p < 0.001], between surveys for Pandemic research, F(1, 797) = 14.492; between surveys for Climate Research F(1, 797) = 19.622, p < 0.001.*

On average, respondents supported more than one policy for each risk, with the exception that in the pandemic survey a majority (64.3%) preferred that governments support none of the three research approaches proposed to address climate change. While it is possible that the solar radiation management and carbon removal policies are more contentious than any of the proposed research for pandemic diseases (on vaccines, tests, and treatments), extensive polling has demonstrated recent strong public support for renewable energy (Howe et al., 2015; Steentjes et al., 2017), which was also one of the climate change mitigation research policy options.

## Discussion

The psychometric risk perception profiles, including moral concerns, for pandemic coronavirus and climate change demonstrate that people see the two risks as similar in many ways. Our results demonstrate that risk perceptions matter; we find that threat and dread form a single dimension in the exploratory factor analysis, as found in much previous work. The extent to which individuals feel informed about the risk and to which they see the risk as understood by science also correlate positively. These two judgments clearly form one dimension. Our confirmatory factor analysis shows that these two judgments load together with the perceived immediacy of consequences on a single factor. However, immediacy had a low loading and was not a reliable component of an additive scale; for these reasons only the first two items were used in the Known scale as a predictor in our regression analyses. In sum, the role of perceived immediacy of risk consequences in the dimension of Known risks is less clear.

A robust result of the regression analyses is that perceived threat is positively and consistently correlated with support for government expenditures on research to reduce risk, for both pandemic coronavirus and climate change, controlling for judgments of efficacy, how well the risk is known, moral concerns and responsibility, political orientation, and demographics.

The idea of the “finite pool of worry” or “worry budget” is that people have limited cognitive capabilities (e.g., Achen and Bartels, 2016), so the emergence of a new, potentially calamitous concern such as pandemic coronavirus must necessarily lead people to worry less about “old” concerns such as climate change. A different view of the consequences of the emergence of a new threat is that the new threat may actually increase overall attention to communal threats as people understand that responding effectively to both these threats requires systemic thinking and cooperative actions at individual, organizational, and national levels. In other words, learning about what needs to be done to control the pandemic coronavirus has a spillover effect of people learning that similarly climate change needs enactment and implementation of new policies. Although we lack the longitudinal data necessary to test these hypotheses, our data definitively demonstrate that people are more likely to support policies that address the threat about which they were encouraged to focus than they are to support policies to address the other threat. The data also show a significant relationship between policy support for the two threats (i.e., people with higher levels of support for policies to address the pandemic coronavirus are more likely also to support policies to address climate change). Perhaps our questions tap three different cognitive realities: a finite pool of worry, acceptance that policy resources are finite, and general support for policies to address communal threats. How people link (or fail to link) their perceptions of the risks from two potentially calamitous threats as well as preferences for policies to address these threats seems to us worthy of extensive further research. The concept of threat fatigue may be a useful addition to future research designs.

A final note of caution regards the survey methods in this study, and the potential threats to validity they pose. The short survey format poses minimal burdens on respondents, and thus is likely to have tapped into spontaneous reactions regarding the risks investigated, pandemic coronavirus and climate change. This can be seen as a positive, to the extent it mitigates response biases, and reduces context biases that might be induced by longer surveys. On the other hand, there is little deliberation, and respondents may not have thought deeply before answering the 10 questions posed to them. To accommodate the short format we implemented a sparse matrix design and used imputation to fill in responses missing completely at random. Imputation methods take full advantage of the information value of the available raw data and are conducted only on responses missing completely at random. Nevertheless, they are less informative than actual responses would be and may underrepresent actual response variability. Another caution is that while the survey is likely representative of internet users in the United States, it is not a true probability sample. Further, although the vast majority of adults in the United States are now internet users, not all are. It follows that the results may be subject to biases stemming from the sampling and survey platform. Finally, we focus here on risk perception in the United States, and these results are not necessarily generalizable to other countries where culture and political attitudes may differ.

This survey provides an empirical snapshot of comparative risk perceptions of pandemic coronavirus and climate change in the initial months of the COVID-19 pandemic in the United States, at a time when comparisons between risks from the pandemic coronavirus and climate change had begun to attract risk analysts’ attention (Bostrom et al., 2020). Further, the study contributes to insights on worry budgets. While this study does not provide within-individual comparative measures of perceived threat, the psychometric results indicate that collectively climate change is still perceived as a threat by the U.S. public, even as the threat of pandemic coronavirus impinges on daily lives. The manifest support for policies to address both pandemic coronavirus and climate change demonstrates that immediate contexts—both the overwhelming presence of the pandemic in April 2020, as well as the immediate pandemic coronavirus survey context—do not completely crowd out concerns about and interests in addressing climate change.

## Data Availability Statement

The raw data supporting the conclusions of this article will be made available by the authors, without undue reservation, to any qualified researcher.

## Ethics Statement

The studies involving human participants were reviewed and approved by University of Washington Human Subjects Committee (IRB ID STUDY00009946). Written informed consent for participation was not required for this study in accordance with the national legislation and the institutional requirements.

## Author Contributions

AB, GB, and RO’C contributed conception and design of the study. AB managed the survey and wrote the first draft of the manuscript. AB, AH, and GB performed the statistical analysis. AH specifically contributing to imputation. GB, RO’C, and AH wrote sections of the manuscript. All authors contributed to iterations of manuscript revision, read and approved the submitted version.

## Conflict of Interest

The authors declare that the research was conducted in the absence of any commercial or financial relationships that could be construed as a potential conflict of interest.
